# Cranberry Juice and Combinations of Its Organic Acids Are Effective against Experimental Urinary Tract Infection

**DOI:** 10.3389/fmicb.2017.00542

**Published:** 2017-04-04

**Authors:** Heidi D. Jensen, Carsten Struve, Søren B. Christensen, Karen A. Krogfelt

**Affiliations:** ^1^Department of Bacteria, Parasites and Fungi, Statens Serum InstitutCopenhagen, Denmark; ^2^Natural Products and Peptides, University of CopenhagenCopenhagen, Denmark

**Keywords:** cranberry juice, urinary tract infection, antimicrobial agents, natural products, *E. coli*, animal model

## Abstract

The antibacterial effect of cranberry juice and the organic acids therein on infection by uropathogenic *Escherichia coli* was studied in an experimental mouse model of urinary tract infection (UTI). Reduced bacterial counts were found in the bladder (*P* < 0.01) of mice drinking fresh cranberry juice. Commercially available cranberry juice cocktail also significantly reduced (*P* < 0.01) bacterial populations in the bladder, as did the hydrophilic fraction of cranberry juice (*P* < 0.05). Quinic, malic, shikimic, and citric acid, the preponderant organic acids in cranberry juice, were tested in combination and individually. The four organic acids also decreased bacterial levels in the bladder when administered together (*P* < 0.001), and so did the combination of malic plus citric acid (*P* < 0.01) and malic plus quinic acid (*P* < 0.05). The other tested combinations of the organic acids, and the acids administered singly, did not have any effect in the UTI model. Apparently, the antibacterial effect of the organic acids from cranberry juice on UTI can be obtained by administering a combination of malic acid and either citric or quinic acid. This study show for the first time that cranberry juice reduce *E. coli* colonization of the bladder in an experimental mouse model of urinary tract infection and that the organic acids are active agents.

## Introduction

Urinary tract infection (UTI), most frequently caused by *Escherichia coli*, is one of the most common bacterial infections in humans (Foxman, 2014). Up to 40–50% of women will suffer from UTI at least once during their lifetime (Foxman, 2003). This high prevalence and the worrying rise in antibiotic resistance among uropathogens emphasize the need for new approaches for treating and preventing UTIs. For many years, cranberry juice has been used as a remedy to prevent and cure UTIs (Jepson et al., 2012; Wang et al., 2012). The beneficial effect of cranberry juice against UTI has been debated in the literature and clinical studies have shown conflicting results; however some studies have shown a protective effect of cranberry juice against UTI (Reviewed by Jepson et al., 2012; Wang et al., 2012; Blumberg et al., 2013; Vasileiou et al., 2013). Cranberry juice is known to inhibit cellular adherence of uropathogenic *E. coli* strains expressing P fimbriae *in vitro* (Ofek et al., 1991; Weiss et al., 1998). Cranberry proanthocyanidins trimers (M_W_ 8–900), were found to abolish *in vitro* adherence of P-fimbriated *E. coli* to cellular structures containing α-Gal (1 → 4) β-Gal binding sites similar to those on uroepithelial cells (Foo et al., 2000a,b). Urine from mice drinking cranberry juice instead of water had bacterial anti-adherence activity *in vitro* (Sobota, 1984). The same activity was observed in urine from mice given drinking water in which proanthocyanidins were dissolved (Howell et al., 2001). This could indicate that a bioactive cranberry proanthocyanidin metabolite was present in the urine preventing adhesion (Howell, 2002). A key characteristic of cranberry juice is the low pH of 2.5 (Hong and Wrolstad, 1986) as well as the unique blend of the organic acids, quinic, malic, shikimic, and citric acid (Jensen et al., 2002), with quinic acid being the most preponderant of the four. The concentration of quinic acid, and the ratio of the concentration of quinic acid to malic acid of 1.36 ± 0.12 are relatively constant (Hong and Wrolstad, 1986; Kuzminski, 1996; Jensen et al., 2002) and are used to calculate percentage of cranberry content in juice drinks and to assess cranberry juice authenticity (Kuzminski, 1996). Major sugars present are glucose and fructose at a glucose-fructose ratio of 3.55, which is unusual for a fruit juice (Kuzminski, 1996). Citric and malic acid are used as food preservatives, being capable of inhibiting a wide variety of microorganisms, including Gram-negative bacteria (Doores, 1993). In addition to acidification, citric acid also functions as a metal-chelating agent (Verhoff, 1986) and may possibly exert its antimicrobial activity by disruptive action on the outer membrane (Helander and Mattila-Sandholm, 2000). Quinic acid might be metabolized to hippuric acid, which is a strong antibacterial agent (Fellers et al., 1933). However, the active compounds responsible for the effect of cranberry juice in UTI has not yet been fully elucidated.

Here, we investigated the antibacterial effect of cranberry juice and the organic acids therein on UTI using a mouse model of long-term ascending urinary tract infection. We show that cranberry juice inhibits *E. coli* bladder colonization and that the organic acids are active agents.

## Materials and methods

### Bacterial strain and preparation of inoculum for infection studies

*E. coli* C175-94, a clinical UTI isolate, was used for the infection studies. It belongs to serotype O8:K48:H9 and express type 1 fimbriae but not P fimbriae (Struve and Krogfelt, 1999). For infection studies, the bacteria were grown overnight at 37°C in static Luria broth (Statens Serum Insitut), centrifuged at 6,500 g for 10 min and the pellet resuspended in PBS (130 mM NaCl, 8 mM Na_2_HPO_4_, 2 mM KH_2_PO_4_, pH 7.4; Statens Serum institut) to a concentration of ~10^10^ CFU/ml.

### Mouse model of ascending UTI

Six- to eight-week old, outbred albino female mice, Ssc:CF1, 30 ± 2 g (SSI) were used. The mice were housed six to a cage and at all times provided free access to feed and water (control group) or treatment solutions. The model used was described in detail previously (Hvidberg et al., 2000). Anesthetized mice were inoculated transurethrally with 50 μl bacterial suspension containing ~5 × 10^8^ CFUs using plastic catheters. The catheter was carefully inserted via the urethral orifice until it reached the top of the bladder and the bacterial suspension was slowly injected into the bladder. The catheter was immediately removed after inoculation and the mice subjected to no further manipulations until sacrifice. The mice were sacrificed 7 days after inoculation. For recovery of bacteria, bladders, and kidneys were aseptically collected in 0.9% saline and homogenized using a sterile grinder (IKA RW16 Basic), and serial dilutions were plated on selective media. All animal experiments were conducted under the auspices of the Danish Animal Experiments Inspectorate, the Danish Ministry of Justice.

### Treatment studies

After inoculation of the mice, the drinking water was substituted with cranberry juice or bioactive compounds in water for the rest of the experiment. A 7 day treatment period was chosen to obtain as long a treatment period as possible but at the same time avoiding the mice cleared the infection spontaneously. The control group and the treated groups all consisted of six mice in each trial. Most of the treatments were repeated two or more times in independent trials and the data were pooled and represented in Table 1. The reproducibility of the control group's infections rates in the independent trials were tested statistically with the Kruskal-Wallis test and found not to vary significantly.

**Table 1:** **Effect on median CFU per g bladder and urinary pH after administration of the specified agents in the drinking water**.

**Treatment**	**Sample**	***n***	**Median (CFU)**	**Interquartile ranges (CFU)**	**Fluid intake/mouse/day (ml ± s.d.)**	**Urinary pH^b^**
Control group (Water)	Bladder	47	5.4 × 10^4^	(3.1 × 10^4^–1.8 × 10^5^)	7.9 ± 2.2	6.5 ± 0.2
Cranberry juice cocktail	Bladder	10	1.9 × 10^4^^*^	(1.5 × 10^4^–8.1 × 10^4^)	4.2 ± 0.3	–
Fresh cranberry juice	Bladder	35	2.9 × 10^4^^**^	(1.6 × 10^4^–6.0 × 10^5^)	3.0 ± 0.5	5.8 ± 0.1
Hydrophilic fraction	Bladder	17	3.0 × 10^4^^*^	(6.3 × 10^3^–6.4 × 10^4^)	3.9 ± 0.3	–
Mixture of organic acids^a^	Bladder	36	2.6 × 10^4^^***^	(1.0 × 10^4^–5.0 × 10^4^)	3.1 ± 0.7	5.8 ± 0.1

a *Mixture of citric, malic, quinic, and shikimic acid in concentrations corresponding to the concentrations found in fresh cranberry juice*.

b *Control n = 14, fresh cranberry juice n = 12; organic acids n = 4*.

*P-values indicated in the table*:

* *P = 0.01–0.05*,

** *P = 0.001–0.01*,

*** *P < 0.001*.

### Cranberry juice and organic acids used for treatment studies

Both commercially available and fresh cranberry juice were tested. Cranberry Juice Cocktail from Ocean Spray® is a 27% single-strength cranberry juice sold in grocery stores. It contains water, high fructose corn syrup, cranberry juice concentrate, and ascorbic acid. Fresh cranberry juice and the preparative isolation of the hydrophilic fraction of cranberry juice were prepared as previously described (Jensen et al., 2002). Frozen American cranberries (*Vaccinium macrocarpon*) from Northland Cranberries® were thawed at 5°C overnight and 1 kg of the berries blended with 700 ml of deionized water for 5 min in a Waring Commercial Blendor. The pulp was centrifuged at 420 g for 15 min and the supernatant was filtered and adjusted to a volume of 1,000 ml fresh cranberry juice. The hydrophilic fraction was made by applying 200 ml cranberry juice on 20 g of RP-18 LiChroprep, 40–63 μm silylated silica gel (Merck) in a 300 × 20 mm column activated with methanol and washed with deionized water. The column was eluted with 300 ml water and the eluates were pooled and evaporated in a vacuum evaporator at 40°C to the volume of the applied juice (200 ml). The major constituents of the hydrophilic fraction were sugars (fructose and glucose), organic acids (quinic, malic, citric, and shikimic acid) and iridoid glycosides (monotropein and 6,7-dihydromonotropein; Jensen et al., 2002). Eluting the column with 96% ethanol (500 ml) gave the ethanolic fraction. The eluate was concentrated to remove the ethanol in a rotavapor (Büchi Rotatvapor-R) at 40°C and kept at −20°C. Before use the residue was redissolved in deionized water to volume corresponding to the applied juice (500 ml). The ethanolic fraction of cranberry juice contained no sugars or organic acids but some not identified compounds, among them anthocyanins.

The organic acids used are commercially available (–)-quinic acid (1β,3α,4α,5β-tetrahydroxycyclohexane carboxylic acid) (Aldrich), L-(–)-malic acid (*S*-hydroxy-succinic acid) (Fluka), (–)-shikimic acid [(–)-3α,4α,5β-trihy-droxy-cyclohexene-1-carboxylic acid] (Aldrich), and citric acid (2-hydroxy-1,2,3-propane-tricarboxylic acid) (Riedel-de Haën). A mixture of the four organic acids, in concentrations corresponding to those found in the hydrophilic fraction of cranberry juice, was tested as well as the acids separately or in mixtures two by two. The concentration of organic acids in the hydrophilic fraction of cranberry juice were 0.74% (w/v) quinic, 0.53% (w/v) malic, 0.03% (w/v) shikimic, and 0.78% (w/v) citric acid, which gave a total concentration of 2.1% (w/v). For the PBS experiment, PBS (130 mM NaCl, 8 mM Na_2_HPO_4_, 2 mM KH_2_PO_4_, pH 7.4; SSI) was pH adjusted to pH 3.0 with HCl.

### Statistical methods

The bacterial counts of infected bladders in each treatment group were compared to the control group using the Mann-Whitney *U*-test. *P* < 0.05 were considered significant. All statistical calculations were performed by use of the GraphPad Prism software.

## Results

### Commercially available and fresh cranberry juice significantly reduce bacterial counts in infected bladders

The mice in the control groups drinking only water were infected with a median of 5.4 × 10^4^ CFU per g bladder at sacrifice day 7 after inoculation (Table 1). Treatment with both commercially available Cranberry Juice Cocktail and fresh cranberry juice reduced the CFU in the bladder by 65% (*P* < 0.01) and 47% (*P* < 0.01), respectively (Table 1). Also treatment with the hydrophilic fraction of cranberry juice decreased CFU in the bladder by 44% (*P* < 0.05; Table 1) whereas treatment with the ethanolic fraction of cranberry juice had no effect. Only very few mice had kidney infection and the bacterial counts were low prohibiting analysis of treatment effect in the kidneys (Results not shown).

### Combinations of the most prevalent organic acids in cranberries has an effect against bladder infection

The most prevalent organic acids from cranberry juice, citric-, malic-, quinic-, and shikimic acid were tested in concentrations corresponding to their respective concentration in fresh cranberry juice. The organic acids were tested as a mixture of four, as well as in combination two by two and individually. Treatment with a mixture of all four organic acids decreased the CFU in the bladder by 52 % (*P* < 0.001; Table 1). Treatment with a combination of malic- and citric acid or malic- and quinic acid decreased the CFU in the bladder by 47% (*P* < 0.01) and 81% (*P* < 0.05), respectively. Treatment with the other combinations of acids namely quinic plus shikimic acid; quinic plus citric acid; citric plus shikimic acid; and malic plus shikimic acid or each organic acid separately had no effect. Also treatment with quinic or citric acid in concentrations corresponding to the total concentration of the four acids did not have an effect.

### Intake of fluid with low pH do not have an effect on bladder infection

To test if oral administration of a fluid with a low pH but without organic acids had any effect, we treated the mice with phosphate buffer pH 3. The treatment did not reduce the bacterial counts of infected bladders (data not shown).

### Mice treated with cranberry juice or solutions of organic acids has lower fluid intake and lower urinary pH

Mice given cranberry juice or solutions of organic acids instead of drinking water drank less than the amount of water consumed by the control group. It could be speculated that the control group drank more due to the infection; however, the amount consumed corresponded with what has been previously described for non-infected mice (Bachmanov et al., 2002). Rather the lower amount consumed by treated mice may be due to the astringent and sour taste of cranberry juice and the organic acids. Importantly, no correlation between fluid intake and reduced CFU could be observed. Thus, addition of quinic acid to the drinking water afforded the lowest consumption of fluid (1.8 ml per day) but had no effect on the number of CFUs, whereas Cranberry Juice Cocktail was consumed in an amount close to pure water (5.8 and 6.5 ml/day, respectively) and significantly reduced the bacterial count in infected bladders. Thus, the observed effect do not appear to be solely related to a more concentrated urine due to a low fluid intake.

The treatments were found to influence urinary pH. Urinary pH was 6.5 in mice drinking water but was lowered to 5.8 in mice drinking cranberry juice or organic acids (Table 1).

## Discussion

The efficacy of treatment with cranberry juice or with constituents in cranberry juice was here, to the best of our knowledge, for the first time investigated in an experimental UTI mouse model. Our results revealed that oral administration to mice of commercially available Cranberry Juice Cocktail, fresh unsweetened cranberry juice, the hydrophilic fraction of cranberry juice, a combination of quinic, malic, citric, and shikimic acid, significantly reduced the number of viable organisms recovered from the bladder (Table 1). The mice consumed more commercially available Cranberry Juice Cocktail than fresh cranberry juice, perhaps due to the higher sugar content in the commercial drink and thereby more palatable taste. This may explain why the treatment effect of the commercial juice were higher than of the fresh juice.

Cranberry juice, have long been used for the prevention and treatment of UTIs. Some clinical studies have shown a prophylactic effect of cranberry juice against UTI in women although conclusions of different studies have been inconsistent (Reviewed by Jepson et al., 2012; Wang et al., 2012; Blumberg et al., 2013; Vasileiou et al., 2013).

Several *in vitro* studies have showed that cranberry juice possess antibacterial (Lee et al., 2000; Puupponen-Pimia et al., 2001; Nogueira et al., 2003), antifungal (Swartz and Medrek, 1968), antiviral (Konowalchuk and Speirs, 1978), and antiadhesive (Sobota, 1984; Schmidt and Sobota, 1988; Zafriri et al., 1989; Ofek et al., 1991; Ahuja et al., 1998; Weiss et al., 1998; Habash et al., 1999; Burger et al., 2000, 2002; Reid et al., 2001) properties.

We investigated the effect of oral administration of a mixture of quinic, malic, shikimic, and citric acid in the concentrations found in cranberry juice. The mixture of organic acids was comparable with cranberry juice in the effect of decreasing the number of CFU in the mouse bladder. The effect of treatment with the organic acids from cranberry juice on CFU in the bladder is interesting since the antibacterial effect of organic acids in cranberry juice have only been sparsely investigated. An effect of oral administered organic acids against diarrhea has been previously described (Tsiloyiannis et al., 2001). Organic acids, among them citric and malic acid were found to have a preventive effect on diarrhea in piglets. Post-weaning diarrhea of piglets is caused mainly by Enterotoxigenic *E. coli* (ETEC) strains (Tsiloyiannis et al., 2001). Groups were compared with regard to the appearance of clinical signs, mortality, weight gain, and feed conversion. All groups supplemented with organic acids had reduced incidence and severity of diarrhea, and performed significantly better than the negative group. The study indicates, that organic acids have an antibacterial and/or anti-adhesive effect on diarrhea causing ETEC strains in piglets (Tsiloyiannis et al., 2001). Our study indicates, that a similar effect on UPEC strains occur in the urinary tract.

In the experimental UTI model, the mice were inoculated directly in the bladder. Therefore, the antibacterial effect must occur at this site and the active components must be excreted in the urine. The organic acids tested in this experiment are likely to be excreted in the urine. Blatherwick and Long observed in 1923 that the excretion of both titratable, organic, and hippuric acid was elevated after the ingestion of cranberries (Blatherwick and Long, 1923). Quinic acid in cranberries might be metabolized to hippuric acid (Quick, 1931; Gonthier et al., 2003), which is a strong antibacterial agent (Fellers et al., 1933). Studies have shown that consuming large amounts of lemon, blackcurrant, and orange juice leads to a higher urinary excretion of citric acid (Wabner and Pak, 1993; Seltzer et al., 1996; Kessler et al., 2002). It is likely, that consumption of large amounts of malic acid would also lead to an increased urinary excretion of this acid.

We measured pH in the urine from infected mice drinking either water or cranberry juice and found a pH of 6.5 ± 0.2 and 5.8 ± 0.1, respectively. Our observation supports previous studies, which reported that cranberry juice functions as a urinary acidifier even in moderate amounts (Blatherwick, 1914; Blatherwick and Long, 1923; Bodel et al., 1959; Kahn et al., 1967; Light et al., 1973; Kinney and Blount, 1979; Schultz, 1984; Jackson and Hicks, 1997; Kessler et al., 2002).

Previous studies have shown that proanthycyanidins in cranberry juice have anti-adhesive effect against P-fimbriated *E. coli* (Foo et al., 2000a,b; Howell et al., 2001). Our study revealed that another antibacterial factor from cranberry juice involved in reducing UTI in a mouse model is the organic acids. The *E. coli* isolate used in the present study did not possess P fimbriae which may explain why the effect of cranberry juice and the combinations of organic acids were comparable. It could be speculated that a relatively higher effect of cranberry juice would be observed against urinary tract infection caused by a P-fimbriated *E. coli* strain. This should be investigated in future studies.

This study is to the best of our knowledge, the first to provide evidence of an antibacterial effect of consumption of cranberry juice and combinations of its organic acids by use of a mouse model of urinary tract infection. The active treatments reduced bacterial counts in the bladder but did not cure the infection, indicating that cranberry juice is not a definite treatment but it may promote clearance of the infection for instance in combination with antibiotics. Future studies designed at investigating the effect of organic acids from cranberries in human UTIs could facilitate the development of a functional food or drink containing these organic acids.

## Author contributions

KK obtained the funding for the project. HJ, SC, and KK designed the study. HJ performed the experimental work. All authors participated in the interpretation of the research data and contributed to the writing of the manuscript.

## Funding

This work was supported by grant 9800 989 from The Danish Medical Research Council to KK.

### Conflict of interest statement

The authors declare that the research was conducted in the absence of any commercial or financial relationships that could be construed as a potential conflict of interest.
